# Supplementary material for: Natural Autoantibodies in Biologic-Treated Rheumatoid Arthritis and Ankylosing Spondylitis Patients: Associations with Vascular Pathophysiology
Source: Int J Mol Sci. 2024 Mar 18;25(6):3429. doi: 10.3390/ijms25063429 (PMC10970856; doi:10.3390/ijms25063429)
Supplement: Supplementary file 1 [file ijms-25-03429-s001.zip › ijms-2894906-supplementary.pdf]

Table S1. Significant correlations between nAAb levels and other parameters.

A. Full (RA+AS) cohort

	BMI-0	CRP-12	RF-0	RF-6	RF-12	FMD-12
**CS IgG-0**	R=0.412 p=0.013					
**TOPO-F4 IgM-0**		R=-0.394 p=0.017				
**TOPO-F4 IgM-12**			R=0.602 p=0.005	R=0.552 p=0.012	R=0.543 p=0.013	
**TOPO-F4 IgG-6**						R=0.364 p=0.029

B. RA subset

	BMI-0	DAS28-12	RF-0	RF-6	RF-12	FMD-0
**CS IgM-0**						R=0.501 p=0.025
**CS IgM-6**						R=0.490 p=0.028
**CS IgM-12**		R=-0.499 p=0.025				R=0.456 p=0.043
**TOPO-F4 IgM-12**			R=0.602 p=0.005	R=0.552 p=0.012	R=0.543 p=0.013	

C. AS subset

	BMI-0	CRP-6	CRP-12	BASDAI-12	FMD-12	PWV-12
**CS IgG-0**	R=0.676 p=0.004					R=-0.495 p=0.047
**CS IgG-6**						R=-0.547 p=0.028
**CS IgG-12**						R=-0.597 p=0.015
**TOPO-F4 IgM-0**			R=-0.624 p=0.010			
**TOPO-F4 IgM-6**			R=-0.501 p=0.048			
**TOPO-F4 IgM 12**		R=-0.575 p=0.020	R=-0.517 p=0.040	R=-0.534 p=0.033		
**TOPO-F4 IgG-6**					R=0.537 p=0.032	
**TOPO-F4 IgG-12**					R=0.724 p=0.002	

The numbers 0, 6 and 12 represent baseline, 6 and 12 months, respectively. Abbreviations: AS, ankylosing spondylitis; BASDAI, Bath Ankylosing Spondylitis Disease Activity Index; BMI, body mass index; CRP, C-reactive protein; CS, citrate synthase; FMD, flow-mediated vasodilation; Ig, immunoglobulin; PWV, pulse-wave velocity; RA, rheumatoid arthritis; RF, rheumatoid factor, TOPO-F4, topoisomerase I fragment 4.
